# Soil core microbial taxa maintain community resistance to drive soil ecosystem multifunctionality under *Alternanthera philoxeroides* invasion

**DOI:** 10.3389/fmicb.2025.1707273

**Published:** 2025-11-06

**Authors:** Xiao Lin, Xinyu Zhang, Shujing Li, Yongming Wang, Haiyang Yu, Wenxiu Qin, Jiaoyang Zhang

**Affiliations:** College of Resources and Environment, Anhui Agricultural University, Hefei, China

**Keywords:** *A. philoxeroides*, microorganisms, soil ecosystem multifunctionality, core microbial taxa, community resistance

## Abstract

Alien plant invasion exerts profound impacts on local ecosystems, like biodiversity, stability, and overall functionality. Understanding the ecological stability of soil microbial communities is essential for elucidating how plant invasions drive changes in soil ecosystem functions. In this study, soil ecosystem multifunctionality (EMF) was examined in relation to the resistance of microbial communities across five *Alternanthera philoxeroides*–invaded sites (Xiaoxian, Hefei, Tongling, Anqing, and Huangshan) within different ecological functional zones in Anhui Province, China, with contrasting soil properties and environmental conditions. Soil samples were collected from invaded and uninvaded plots, microbial core taxa were identified, and structural equation modeling was applied to examine the relationships among invasion, microbial traits, and ecosystem functions. The results showed that invasion significantly increased soil EMF at Xiaoxian by 34.2% and at Hefei by 24.1%, which was primarily by increasing in nitrogen- and phosphorus-related functions. Bacterial communities exhibited consistently higher resistance than fungal communities, owing to their more complex interaction networks. Core bacterial taxa were positively associated with microbial resistance, supporting nutrient cycling and maintaining ultimately EMF. Structural equation modeling revealed that invasion-driven changes in soil properties influenced EMF indirectly through bacterial community resistance mediated by core microbial taxa. These findings demonstrated that bacterial resistance, underpinned by the stabilizing role of core microbial taxa, played a central role in maintaining EMF during plant invasion. The results highlight the importance of core microbial taxa as ecological stabilizers and provide new insights into the belowground mechanisms linking biological invasions to ecosystem resilience.

## 1 Introduction

Invasive alien plants have attracted increasing global attention due to their profound impacts on terrestrial biodiversity and stability (Lai et al., 2025). These impacts extend to key ecosystem processes, like biogeochemical cycling. For instance, the invasion of *Ligustrum* in the Andes significantly accelerated biomass accumulation in successional forests, thereby enhancing carbon sequestration (Nguyen et al., 2020). At a broader scale, a global meta-analysis revealed that invasive plants could exhibit higher rhizosphere nitrogen mineralization rates, greater soil available nitrogen content, and increased activity of nitrogen-releasing enzymes compared with native species, ultimately enhancing soil nitrogen cycling (Negesse et al., 2025). In addition to altering carbon and nitrogen dynamics, invasive species could influence phosphorus turnover. For example, *Bidens pilosa* showed approximately 30% higher alkaline phosphomonoesterases activity in the rhizosphere than native plants, directly promoting the decomposition of organic phosphorus (Sun et al., 2022). While such studies provide valuable insights into individual soil element cycling function, they often overlook the reality that ecosystems operate through multiple, interconnected and trade-off functions. Understanding how these functions occur simultaneously is essential for capturing the full complexity of ecosystem responses. Collectively, biogeochemical cycling serves as a fundamental basis for ecosystem functions, underpinning processes such as productivity, nutrient turnover, and carbon sequestration. EMF provides an integrative framework to evaluate these processes, enabling a more comprehensive assessment of invasion consequences by capturing both trade-offs and synergies among ecosystem functions. This system-level perspective not only links community structural changes with ecosystem processes but also offers valuable guidance for ecosystem management and restoration under invasion pressure. Yet, comprehensive evaluations of EMF that incorporate multiple, interrelated soil functions are still lacking, leaving the ecosystem-level consequences of plant invasion poorly understood. Addressing this knowledge gap, the present study examines how plant invasion affects soil EMF by integrating multiple biogeochemical and microbial properties, thereby providing a comprehensive assessment of ecosystem responses to invasion.

Soil microbial communities, with their highly complex species composition, are sensitive and adaptable to environmental change due to traits such as metabolic flexibility, large population sizes, and rapid adaptation (Allison and Martiny, 2008). These attributes underpin their resistance, the capacity to maintain structural, compositional, and functional stability under stress (Shade et al., 2012), a key aspect of ecosystem stability. However, resistance can vary with disturbance intensity and environmental context, and may be altered by global change drivers such as climate, land use, and nutrient availability (Jiao et al., 2022; Zheng et al., 2025; Zhou et al., 2020). Plant invasion represents a major biotic disturbance that can reshape microbial stability and community composition. Invasive plants influence soil microorganisms by plant secondary metabolites or changes in higher trophic interactions, referring to resource-driven influences such as enhanced nutrient acquisition and root exudation (Xiao et al., 2014; Zhang et al., 2020). These processes can accelerate bacterial community succession, reduce stochasticity in community assembly, and lead to uneven shifts in microbial composition (Zhao et al., 2024). For example, invasive plants enhance nitrogen turnover through interactions with rhizosphere microorganisms, thereby strengthening their competitive advantage (Ji et al., 2024). And the invasion of *Mikania micrantha* has been shown to alter microbial metabolic functions in subtropical forests, creating conditions more favorable for its own growth (Zhao et al., 2023). Yet, it remains unclear how such invasion-driven changes in microbial resistance influence soil ecosystem functions.

Most research on invasion–microbe interactions have emphasized overall diversity or community composition, while largely overlooking the contributions of specific functional taxa, which directly underpin key ecosystem processes. Microbial communities are structured into distinct taxa with differing ecological importance, among which core and keystone microbial taxa are particularly critical for maintaining function and network stability (Jacquiod et al., 2020). These taxa are consistently present across diverse habitats, often exhibit stable genetic traits, contribute disproportionately to multiple microbial functions (Jiao et al., 2021; Shade and Handelsman, 2012; Sun et al., 2024), and occupy broader ecological niches (Scheller and Parajuli, 2018; Vietorisz et al., 2025), thereby enhancing ecosystem resilience (Coban et al., 2022; Jiao et al., 2022). Alterations in these taxa may play a pivotal role in mediating the impacts on EMF. For example, shifts in arbuscular mycorrhizal fungi, a keystone microbial taxa in farmland and saline-alkali wasteland ecosystems, can alter soil nutrient supply (Zhu et al., 2025), while changes in denitrifying or cellulose degrading bacteria can influence nitrogen retention and organic matter turnover (Hu et al., 2019; Luo et al., 2020), ultimately affecting both the resistance and functional stability of soil ecosystems. Nevertheless, empirical evidence from invasive ecosystems remains limited (He et al., 2025). Introducing the perspective of core microbial taxa into invasion ecology is therefore novel, as it moves beyond documenting diversity loss or compositional shifts to reveal the stabilizing mechanisms that buffer invasion-induced disturbances. Unlike agricultural or forest ecosystems, where core taxa are often studied in relation to long-term management practices or successional stability, invaded ecosystems provide a unique context in which core taxa mediate sudden, non-equilibrium disturbances caused by exotic plants, thereby shaping soil EMF. Understanding how core and keystone microbial taxa respond to invasion and contribute to EMF is therefore essential for elucidating the mechanisms that link above- and belowground processes.

To address this gap, we focused on the perennial invasive species *Alternanthera philoxeroides*, which can alter soil ecosystem processes through complex interactions with soil microbial communities (Fan et al., 2016). Plant and soil samples were collected from abandoned fields (>2 years) that had been invaded by *A. philoxeroides*, together with adjacent non-invaded fields serving as controls. Through integrated analyses, we aimed to combine invasion, changes in EMF, and shifts in core microbial taxa. Specifically, we tested the following hypotheses: (1) *A. philoxeroides* invasion enhances soil EMF; (2) core and keystone microbial taxa, particularly bacteria, are critical for maintaining soil microbial stability in response to invasion; (3) the core microbial taxa and the resistance they mediate are closely related to the improvement of soil EMF. Clarifying these relationships is essential for understanding the mechanisms by which biological invasions reshape belowground ecosystems and for informing strategies to preserve or restore soil EMF under global change.

## 2 Materials and methods

### 2.1 Site description

**Figure 1:**
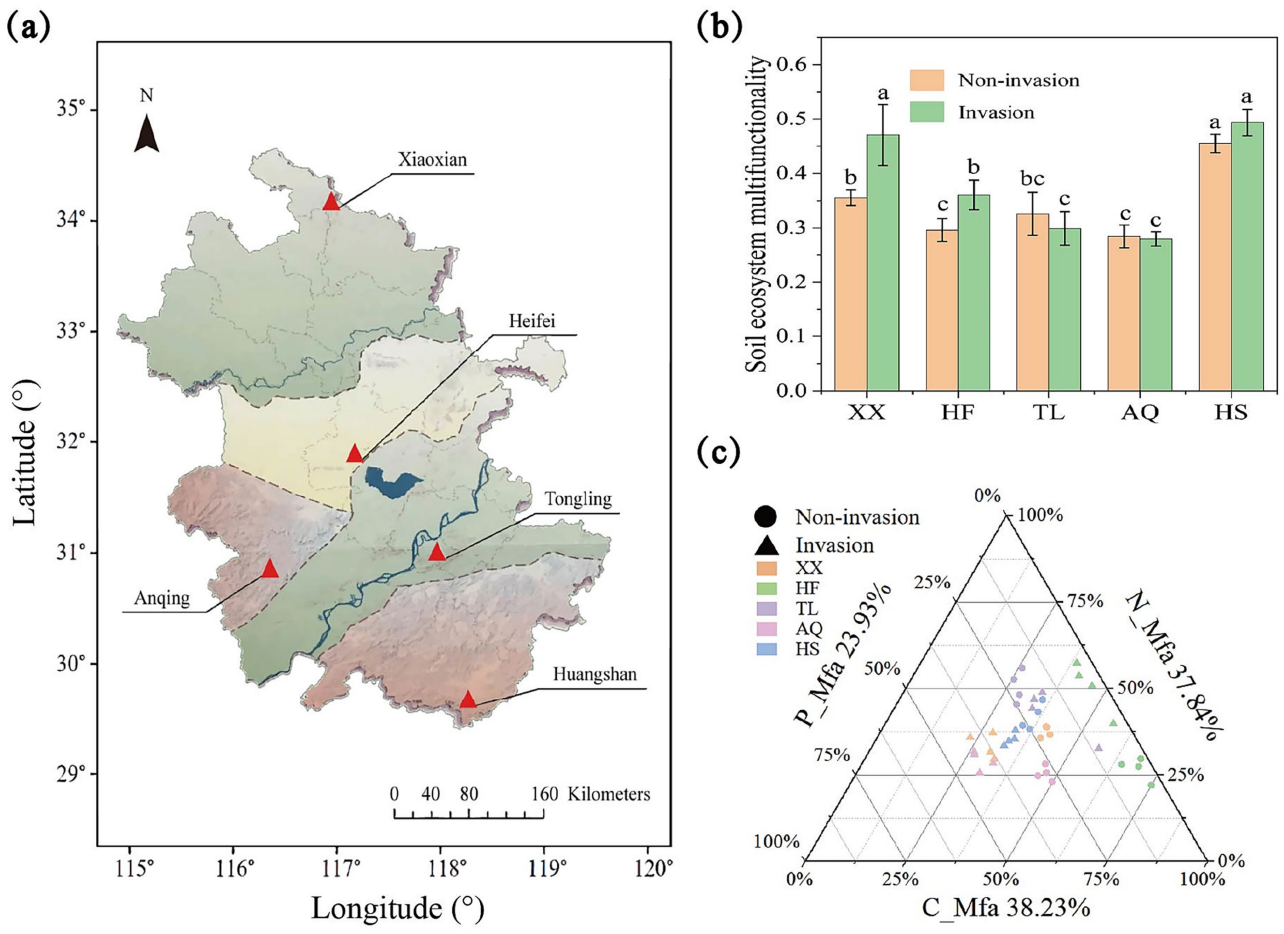
The changes of soil functional indicators after the invasion of *Alternanthera philoxeroides*. **(a)** The position information of different sites; **(b)** The histogram of the effect of *A. philoxeroides* invasion on soil multifunctionality; **(c)** The ternary phase diagram of C, N, P cycling function of *A. philoxeroides* invasion background soil ecosystem, all functional values were standardized using the “*Z*-score” method.

To maximize regional representativeness, we selected the Yangtze–Huaihe River Basin, a north–south climatic transition zone with pronounced edaphoclimatic heterogeneity. The study area spans five ecological sub regions: (i) The Plains Ecological Zone north of the Huaihe River, (ii) The Hilly and Ridge Ecological Zone between the Yangtze and Huaihe Rivers, (iii) The Dabie Mountain Ecological Zone in western Anhui, (iv) The Yangtze River Plain Ecological Zone, and (v) The Southern Anhui Mountainous and Hilly Ecological Zone. Based on prior work and field reconnaissance, we confirmed that *A. philoxeroides* are widely invasive across these zones. The locations of the sampling sites are shown in Figure 1a, and their edaphoclimatic contrasts are summarized in supporting information.

Xiaoxian, located in a warm-temperate semi-humid monsoon climate and dominated by sandy soil, had the lowest mean annual precipitation (MAP, 854 mm) and mean annual temperature (MAT, 14.3 °C). Hefei lies in a subtropical humid monsoon climate with yellow-brown soils and received 1,000 mm MAP, with a MAT of 15.7 °C. Tongling, situated in a mid-subtropical humid monsoon climate dominated by yellow-brown and brownish-red soils, recorded 1,412 mm MAP and a MAT of 15.9 °C. Anqing, positioned at the transition between subtropical humid and warm-temperate semi-humid zones, with yellow-brown, yellowish-red, and mountain meadow soils, received 1,427 mm MAP and had a MAT of 14.5 °C. Huangshan, located in the transition from northern to central subtropical climate and characterized by red, yellow, dark yellow-brown, and mountain meadow soils, recorded the highest MAP (1706 mm) with a MAT of 16.1 °C.

### 2.2 Experimental design and soil samples collection

After finalizing the sampling sites, we conducted fieldwork in September–October 2022 across five ecological zones. In each zone, we established four paired sites—*A. philoxeroides*–invaded versus uninvaded controls—on land that had been abandoned for >2 years (The land had been used for tillage before being abandoned, after which *A. philoxeroides* began to invade). Each sample site was no more than 30 m between the *A. philoxeroides* invasion-affected and non-invasion-affected sample plots to minimize the effects of soil background differences. Four pairs of sample plots were separated by a distance of no less than 50 m. Each sample plot was 1 × 1 m^2^ and was used for investigating the basic characteristics of the vegetation and soil sample collection. The aboveground portions of plants within each small quadrat were harvested at ground level and sorted by species. Each species was then stored separately in envelopes and brought back to the laboratory for the determination of vegetation aboveground biomass. Soil samples from 0 to 20 cm depth were collected at multiple points in an S-shaped pattern within each quadrat. Soil samples from the same quadrat were mixed, and stones, plant roots, litter, and visible soil fauna were removed. The fresh soil was then divided into two portions. One portion was air-dried and sieved through a 0.25 mm sieve for the determination of basic soil physicochemical properties. The other fresh sample was sieved through a 2 mm sieve and further divided into two subsamples. One of these subsamples was stored in a 4 °C refrigerator for the measurement of microbial biomass carbon, nitrogen, and phosphorus, soluble organic carbon, mineralization rate, soil phosphatase, β-glucosidase, and N-acetyl-β-glucosidase. The other subsample was stored in a −80 °C freezer for DNA extraction followed by microbial community characterization by marker gene analysis.

### 2.3 Determination of soil physical and chemical properties

Soil samples were analyzed for various parameters using specific methods. The potassium dichromate oxidation-external heating method was used to determine soil organic carbon (SOC) content (Nóbrega et al., 2015), the Kjeldahl method for total nitrogen (TN) (Bremner and Mulvaney, 1982), as well as the sodium hydroxide alkali fusion-molybdenum antimony colorimetric method for total phosphorus (TP) contents (Schade et al., 2003), respectively. To measure soil ammonium nitrogen (NH_4_^+^–N) and nitrate nitrogen (NO_3_^−^–N) content, extraction was performed using a KCl solution (1 mol·L^−1^). Soil pH was determined using solutions with a ratio of 1:2.5 (soil:water), the pH electrode type is a glass electrode, and the calibration temperature is 25 °C. Soil nitrogen mineralization rate (NMR) was determined by anaerobic method (Morecroft et al., 1992). Soil available phosphorus (AP) was extracted using NaHCO_3_ and measured with a spectrophotometer (Olsen, 1954). Additionally, Soil microbial biomass, including soil microbial biomass carbon, nitrogen, phosphorus (MBC, MBN, MBP), was determined by chloroform fumigation method (Harris et al., 1997; Oberson et al., 1997).

### 2.4 Soil enzymatic activity analysis

In this study, soil enzyme activity analysis included soil phosphatase, soil β-glucosidase (S-β-G-C), and soil N-acetyl-β-D-glucosidase (NAG). All of them were detected by Beijing Sheng gong soil enzyme activity detection kit, the unit of enzyme activity is U/g (1 μmol of p-nitrophenol is produced per gram of soil every day). Soil phosphatase activity detection method: 0.1 g soil sample was taken in 2 mL centrifuge tube, 0.05 mL toluene was added, 15 min later, substrate was added, and 24 h was catalyzed in 37 °C environment. After mixing and centrifugation, the supernatant was added to 96 microporous plates, and the absorbance value was measured at 660 nm wavelength by multifunctional microplate reader, and the enzyme activity index was calculated according to the standard curve. Soil β-glucosidase and soil N-acetyl-β-D-glucosidase detection method, 30 mg of soil was taken in a 1.5 mL centrifuge tube, and the corresponding substrates were added, respectively. The reaction was catalyzed at 37 °C for 1 h, and then immediately taken out and placed in a boiling water bath for 5 min. The process must ensure that the centrifuge tube is closed. After natural cooling, the supernatant was centrifuged and added to a 96-well microplate. The absorbance was measured at a wavelength of 400 nm using a multifunctional microplate reader, and the enzyme activity index was calculated according to the standard curve. In the above method, each soil sample needs to be set up a control group and a blank group.

### 2.5 Microbial community analysis

To isolate microbial DNA from soil samples, 0.25 g of soil were processed using the TIANamp Soil DNA Kit (Tiangen Biotech Co., Ltd., Beijing, China). The purity and concentration of the extracted DNA were rigorously evaluated using a spectrophotometer. For the purpose of amplifying bacterial and fungal genes, the specific regions of interest were the V3–V4 region of bacterial genes and the ITS1 region of fungal genes. A polymerase chain reaction (PCR) was conducted with tailored primers: 341F/806R (Wang et al., 2019) for bacteria and 1737F/2043R (Hao et al., 2016) for fungi. The PCR reaction mixture was composed of 2 μL of sterile ultrapure water, 15 μL of Phusion Master Mix (2× concentration), 3 μL of 6 μM primers, and 10 μL of template DNA (ranging from 5 to 10 ng). The amplification protocol initiated with a denaturation step at 98 °C for 60 s, followed by 30 cycles, each comprising denaturation at 98 °C for 10 s, annealing at 50 °C for 30 s, and extension at 72 °C for 30 s. A final extension step was executed at 72 °C for 5 min. Subsequently, the PCR products were purified with a Qiagen Gel Extraction Kit (Qiagen, Germany) and subjected to sequencing on the Illumina HiSeq2500 platform (Majorbio Bio-Pharm, Shanghai).

The raw sequence data underwent rigorous processing and chimera screening utilizing QIIME (Caporaso et al., 2010). During this step, chimeric sequences were identified and excluded, while the remaining sequences were clustered into operational taxonomic units (OTUs) based on a 97% similarity threshold using UPARSE (Edgar, 2013). Taxonomic assignment of the OTUs was then performed utilizing the Green Genes Database 13_5 and the RDP classifier (DeSantis et al., 2006).

### 2.6 Calculation of EMF

In assessing the functional indices of soil ecosystems, we selected 14 ecosystem functional indices modulated by soil microorganisms. These were consolidated into three primary functional indices pertaining to soil carbon, nitrogen, and phosphorus cycling, respectively. Specifically, the soil C multifunctionality (C_Mfa) indices encompassed SOC, DOC, S-β-G-C, and MBC. Similarly, the soil N multifunctionality (N_Mfa) indices comprised TN, NH_4_^+^–N, NO_3_^−^–N, NMR, NAG, and MBN. Lastly, the soil P multifunctionality (P_Mfa) indices included TP, AP, MBP, and SAP. Additionally, soil pH, soil moisture content (SMC), and aboveground biomass (AGB), which are recognized as factors influencing soil ecosystem function, were measured and subsequently subjected to a joint analysis alongside the multifunctional indices of the ecosystem. Prior to the calculation of emf, all functional values were standardized using the “*Z*-score” method, i.e., standardized using the mean and standard deviation (measured values minus the mean divided by the standard deviation) (Maestre et al., 2012). Afterward, the multifunctionality index *MF_a_* was calculated using the following formula: according to the following formula:


$${\mathrm{MF}}_{a}=\frac{1}{F}\sum_{i=1}^{F}g(r_{i}(f_{i}))$$

Here, *F* refers to the amount of functions determined; *f_i_* refers to the determined value of function *i*; *r_i_* is a mathematical function that converts *f_i_* into a positive value; and *g* refers to the standardization of all determined values.

### 2.7 Statistical analysis

Microbial community resistance is typically defined as the extent to which microorganisms exhibit tolerance in response to disturbances. In this study, microbial community resistance was specifically quantified by assessing the similarity of microbial communities between invaded and non-invaded sites within each ecological zones, utilizing the “vegan” package in R 4.3.1 for data processing.

Microbial network diagrams can assist us in better observing the changes in microbial community structure in response to disturbances. By screening the top 500 species at the genus level of bacteria and fungi, we can select OTUs, species, genera, and phyla with significant interaction effects, as well as the interaction information among them. The data are then processed in Gephi 0.9.7 for visual analysis of the interaction relationships within the microbial community.

Utilizing R, the raw OTU files of bacteria and fungi are processed to identify eligible microbial functional groups. Core microbial taxa are selected based on OTUs that account for the top 10% of abundance across all samples and are ubiquitously present in 50% or more of soil samples, thus recognized as core microbial taxa. Key taxa are identified by first calculating the Spearman rank correlation coefficients among OTUs to form a relationship matrix, followed by selecting OTUs with absolute correlation coefficients ≥ 0.8, along with their corresponding genera and phyla, which are then recognized as key taxa.

Mantel analysis facilitates a deeper understanding of the relationships between different microbial functional groups and environmental factors. Mantel test was conducted in R using the “linkET,” “ggplot2,” and “dplyr” packages, and the results were visualized graphically. Performing regression analysis between microbial communities and ecosystem functions, facilitated by SPSS 22 for data processing and Origin 2019 for plotting regression models, can enhance our understanding of the correlations between individual communities and specific ecosystem functions. Furthermore, structural equation modeling (SEM) assesses the linkages among environmental factors, soil microorganisms, and ecosystem functions, involving data processing in SPSS 22 and subsequent model construction in SPSS Amos 26.

## 3 Results

### 3.1 Response of soil EMF and elemental cycling related function effects of *A. philoxeroides* invasion

Overall, the invasion of *A. philoxeroides* exhibited region-specific effects on soil EMF (Figure 1b). EMF increased significantly in Xiaoxian and Hefei after invasion, whereas changes in Tongling, Anqing, and Huangshan were not significant. Trade-off analysis of C-, N-, and P-related functions revealed that carbon- and nitrogen-associated multifunctionality (C_Mfa and N_Mfa) dominated overall EMF, contributing 38.23 and 37.84%, respectively, while phosphorus-associated multifunctionality (P_Mfa) accounted for 23.93% (Figure 1c). Most samples were distributed along a balanced gradient between C_Mfa and N_Mfa. Invasion did not act uniformly but instead rebalanced functional contributions along the C–N–P axes. In Xiaoxian and Anqing, C_Mfa shifted toward P_Mfa; in Hefei, it shifted toward N_Mfa; in Huangshan, N_Mfa shifted toward P_Mfa; and in Tongling, invaded and uninvaded samples remained closely clustered, reflecting a comparatively balanced functional structure.

### 3.2 Responses of microbial community and microbial taxa to *A. philoxeroides* invasion

**Figure 2:**
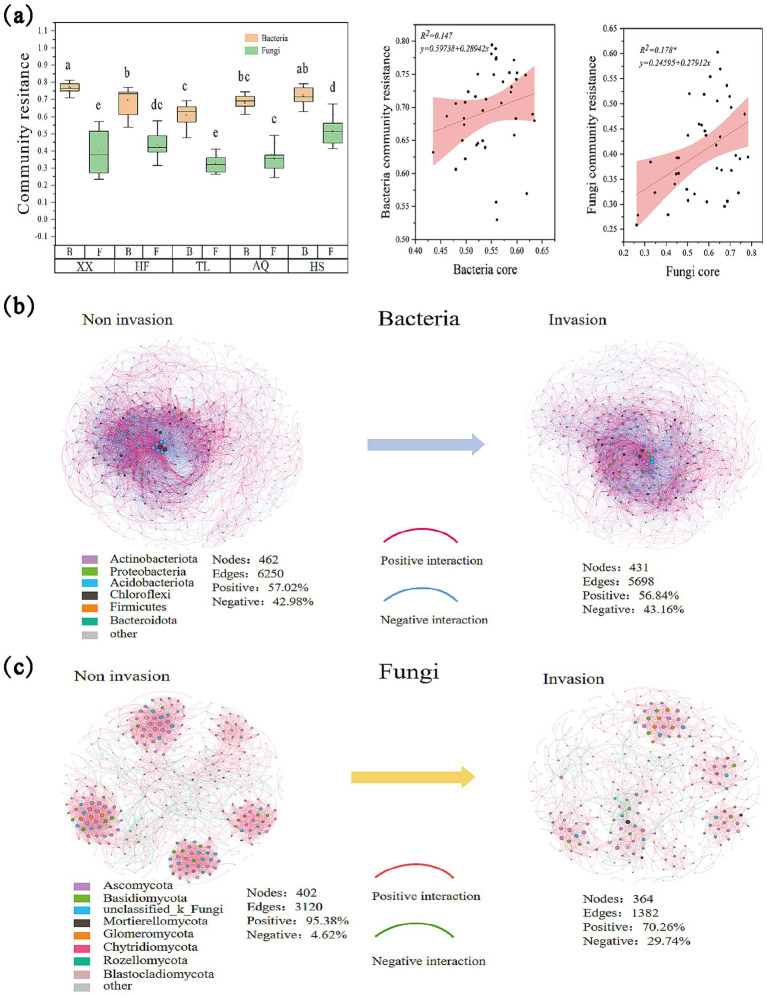
Changes in bacterial and fungal communities. **(a)** The difference in resistance between soil bacterial and fungal communities; The regression analysis between bacterial community resistance and EMF; **(b)** The interaction effect of species in the bacterial community during *A. philoxeroides* invasion, in which the nodes are labeled with different colors at the gate level, and the information of the corresponding phylum is labeled in the lower left corner of the network diagram. The pink connection line indicates a positive interaction, and the blue connection line indicates a negative interaction. Nodes represent the number of nodes, Edges represent the number of connection lines, Positive represents the positive interaction rate, and Negative represents the negative interaction rate, the same below; **(c)** The interaction effect of species in the fungal community during *A. philoxeroides* invasion, in which the nodes are labeled with different colors at the gate level, and the information of the corresponding phylum is labeled in the lower left corner of the network diagram. And the pink connection line indicates positive interaction and the green connection line indicates negative interaction.

Community resistance analysis showed that bacterial communities exhibited significantly higher resistance than fungal communities across all regions (Figure 2a). For bacteria, community resistance differed significantly between Xiaoxian, Hefei, and Tongling regions with Anqing and Huangshan regions, showing a decreasing trend with increasing latitude. For fungal communities, the highest resistance was observed in Huangshan, which was significantly greater than in Xiaoxian, Tongling, and Anqing, but not significantly different from Hefei.

In the co-occurrence networks constructed from the 500 most abundant OTUs, the bacterial networks included phyla such as Actinobacteriota, Proteobacteria, Acidobacteriota, Chloroflexi, and Firmicutes, and the fungal networks included Ascomycota, Basidiomycota, Mortierellomycota, and unclassified fungi (Figures 2b,c). For bacteria, the pre-invasion network contained 462 OTUs and 6,250 edges (57.02% positive, 42.98% negative), which decreased to 431 OTUs and 5,698 edges (56.84% positive, 43.16% negative) after invasion. For fungi, the pre-invasion network contained 402 OTUs and 3,120 edges (95.38% positive, 4.62% negative), which decreased to 364 OTUs and 1,382 edges (70.26% positive, 29.74% negative) after invasion.

**Figure 3:**
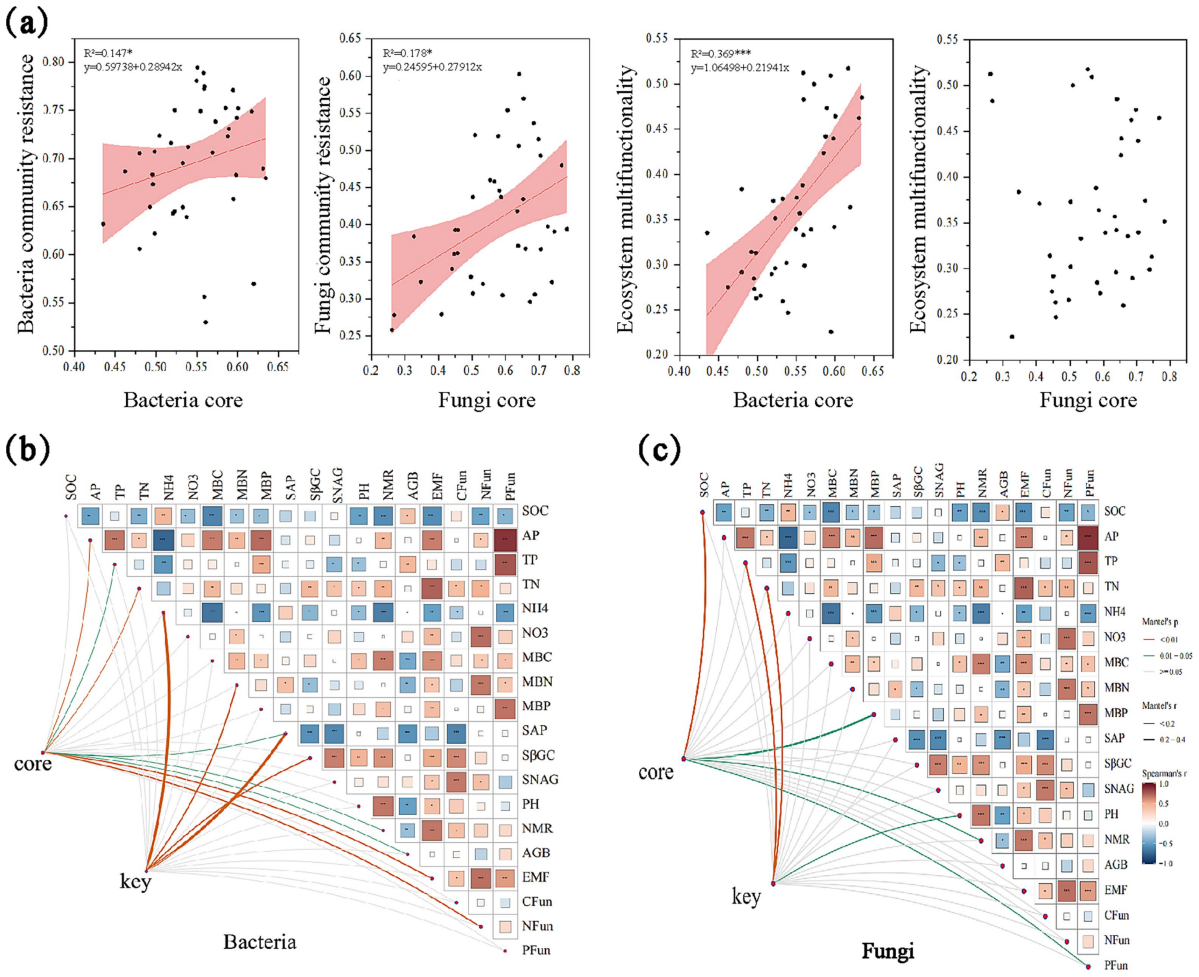
Linking bacterial and fungal core, key taxa and ecosystem multifunctionality. **(a)** The regression analysis between bacterial and fungi core, key taxa and environmental factors; **(b)** The correlation between bacterial core taxa and community resistance and EMF. Line width corresponds to the partial Mantel’s *r* statistic. Pairwise comparisons of environmental factors are also shown, with a color gradient denoting Pearson’s correlation coefficient. Asterisks indicate the statistical significance (***p < 0.001; **p < 0.01; and *p < 0.05), the same below; **(c)** The correlation between fungal core taxa and community resistance and EMF.

Mantel tests indicated that core bacterial taxa were significantly correlated with soil available phosphorus (AP), total nitrogen (TN), EMF, and N_Mfa (*p* < 0.001), and also related to total phosphorus (TP), phosphatase activity, nitrogen mineralization rate (NMR), and aboveground biomass (AGB) (*p* < 0.05) (Figure 3b). Keystone bacterial taxa were strongly correlated with ammonium nitrogen (NH₄^+^–N), microbial biomass nitrogen (MBN), phosphatase activity, and soil β-glucosidase activity. In the fungal community, core microbial taxa were strongly associated with soil organic carbon (SOC) and significantly correlated with microbial biomass phosphorus (MBP), NMR, and P_Mfa. Keystone fungal taxa were significantly related to TP, TN, and soil pH (Figure 3c).

### 3.3 Community resistance mediated by microbial taxa drives changes in soil EMF

**Figure 4:**
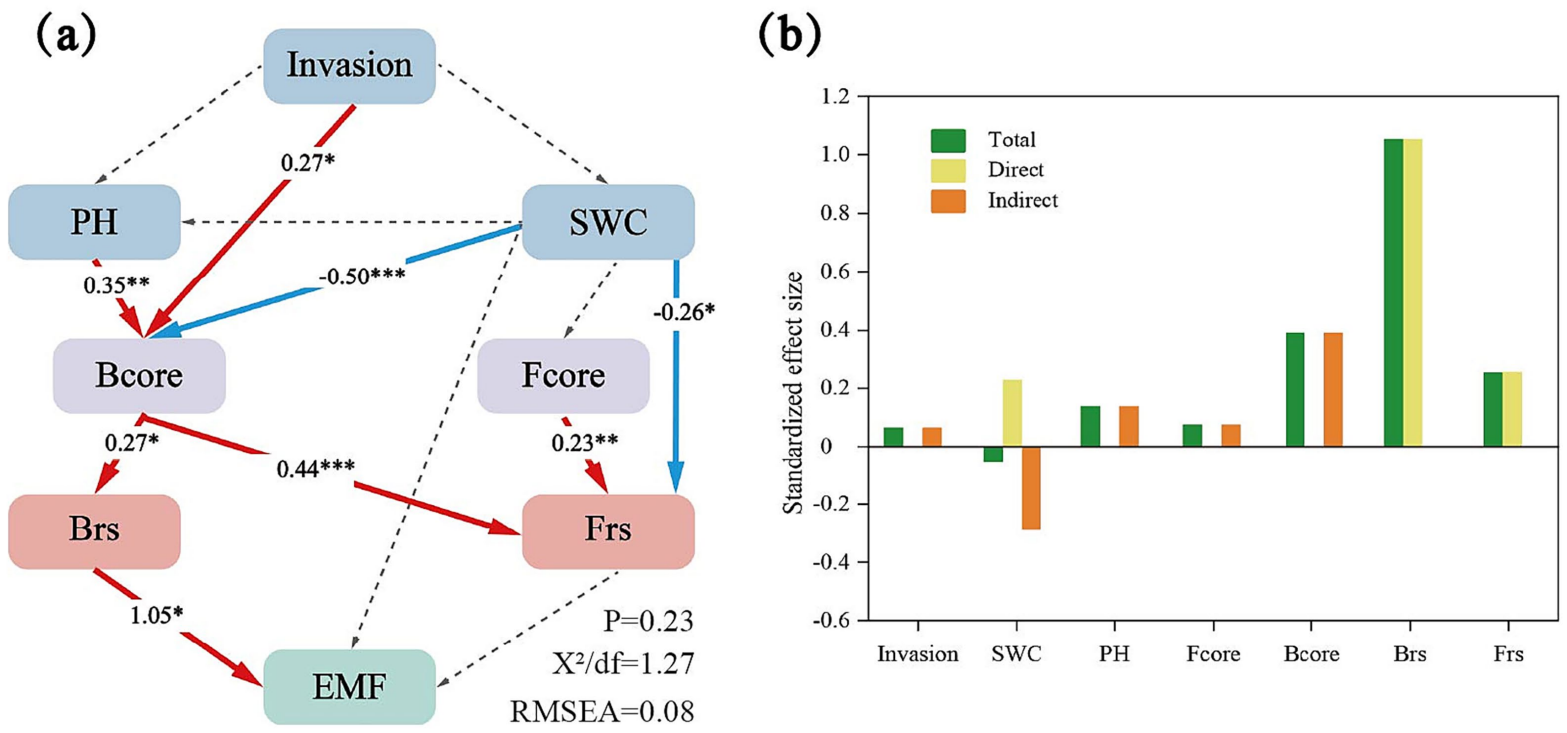
**(a)** Structural equation modeling (SEM) assessing the effects of Invasion, PH, SWC (soil water content), B and Fcore (bacterial and fungal core taxa), B and Frs (bacterial and fungal community resistance) in EMF (ecosystem multifunctionality). Numbers adjacent to arrows show standardized path coefficients. Red and blue lines indicate positive and negative relationships. ***p < 0.001; **p < 0.01; and *p < 0.05. **(b)** Standardized total, direct and indirect effects based on the SEM for the concentrations of Invasion, PH, SWC, B and Fcore, B and Frs. The green column is the total effect, the yellow column is the direct effect, and the orange column is the indirect effect.

Furthermore, both bacterial and fungal community resistance were positively associated with EMF (Figure 2a), indicating that higher microbial resistance was associated with enhanced EMF. Significant positive correlations were observed between selected core microbial taxa (both bacterial and fungal taxa) and community resistance (Figure 3a). Structural equation modeling (SEM) further clarified these relationships (Figure 4, Structural equation modeling overall fit indices: CFl = 0.96, TLI = 0.90, RMSEA = 0.08, SRMR = 0.02, *x*^2^/*df* = 1.27). Invasion of *A. philoxeroides* and changes in soil properties significantly affected the abundance of core bacterial taxa. Soil water content exerted a strong negative effect on these taxa (path coefficient = −0.50, *p* < 0.001). The core bacterial taxa were positively associated with the resistance of both bacterial and fungal communities. Notably, bacterial community resistance had a direct and significant positive effect on soil EMF (path coefficient = 1.05, *p* < 0.05), indicating that the stabilization of microbial communities played a substantial role in maintaining EMF.

## 4 Discussion

### 4.1 Impact of *A. philoxeroides* invasion on soil EMF

Soil EMF is a comprehensive indicator of the soil’s capacity to perform multiple ecosystem services and functions simultaneously, supporting nutrient cycling, carbon sequestration, water regulation, biodiversity maintenance, and pollutant degradation (Delgado-Baquerizo et al., 2016; Liu et al., 2023). Our results showed that the invasion of *A. philoxeroides* significantly enhanced soil EMF in both Xiaoxian and Hefei sites (Figure 1b), with nitrogen and phosphorus cycling being particularly affected. Multifunctionality trade-off analyses for carbon, nitrogen, and phosphorus revealed that in Xiaoxian, the invasion increased the proportion of P_Mfa, whereas in Hefei, it enhanced N_Mfa, both contributing to elevated soil EMF (Figure 1c). However, unlike the general consensus that plant invasions often suppress soil functions (Negesse et al., 2025), *A. philoxeroides* invasion appears to promote soil EMF. This outcome likely reflects the combined influence of plant traits, soil properties, and environmental conditions (Hu et al., 2024). In Xiaoxian, lower MAP, sandy soil and *A. philoxeroides*’s complex root network create a loose soil texture (Li et al., 2009). Improved aeration accelerates organic matter decomposition (Paul, 2016), releasing phosphorus bound in organic matter (Feng et al., 2022; Yang et al., 2022), which enhances phosphorus turnover and elevates EMF. In Hefei, the strong stress tolerance, rapid proliferation, and extensive root system of *A. philoxeroides* facilitate root secretions that provide carbon sources for rhizosphere nitrogen-fixing bacteria (Li et al., 2023; Yang et al., 2020). This symbiotic interaction promotes nitrogen cycling, further increasing EMF. These region-specific pathways suggest that elevated soil EMF is regulated by differential nutrient turnover processes across sites. Given that soil microorganisms are central to nutrient cycling and biogeochemical regulation, invasion-induced microbial restructuring likely mediated these effects (Long et al., 2025; Xu et al., 2025). While invasive plants often reduce microbial diversity and network stability (Chen et al., 2019; Yin et al., 2020), the core microbial taxa associated with *A. philoxeroides* are robust to such disturbances and can functionally reconfigure soil nutrient dynamics. Taken together, our findings highlight that the enhancement of soil EMF by *A. philoxeroides* results from the interplay between altered soil properties and microbial community restructuring, supporting our first hypothesis.

### 4.2 Effects of *A. philoxeroides* invasion on core-taxa-driven microbial resistance

Our results showed that bacterial communities exhibited stronger resistance than fungal communities across regions (Figure 2a), consistent with previous findings (Delgado-Baquerizo et al., 2016; Jiao et al., 2022). This difference likely reflects contrasting ecological strategies: bacteria, with denser interaction networks and greater interspecies connectivity (Figure 2b), form cooperative and functionally versatile assemblages (Wang et al., 2022; Zhang et al., 2018). These relationships, coupled with high metabolic flexibility, enable rapid adjustment to environmental fluctuations (Wang et al., 2022), enhancing resistance to invasion-driven disturbances. In contrast, fungi often occupy narrower niches and rely on more specialized resource-use strategies, which slow their responses to change and reduce short-term contributions to community resistance (Chen et al., 2020; Jing et al., 2015).

The capacity of microbial communities to resist invasion is closely linked to the presence and function of core microbial taxa, which are consistently detected across habitats and disproportionately shape community structure and ecosystem processes (Jacquiod et al., 2020; Sun et al., 2024). In our study, these taxa were positively correlated with microbial resistance (Figure 3a), supporting their role as stabilizing agents within microbial networks (Wertz et al., 2007; Zhu et al., 2016). Mechanistically, their stabilizing effect likely arises from three complementary traits: stable diversity, which maintains functional groups across gradients; functional redundancy, which buffers against loss of individual taxa; and niche complementarity, which promotes efficient resource partitioning and minimizes competition. Together, these mechanisms explain why core microbial taxa are pivotal in maintaining microbial community stability under invasion pressure, supporting our second hypothesis.

### 4.3 Core microbial taxa mediating microbial resistance as a driver of soil EMF

Our results revealed a positive relationship between bacterial community resistance and soil EMF (Figure 2a), consistent with previous findings that microbial resistance benefited ecosystem functions. This relationship stems from the capacity of microbial communities to maintain compositional and functional stability, thereby supporting ecosystem processes. In this study, bacterial communities exhibited broad functional stability owing to their complex interaction networks, particularly within core microbial taxa such as Proteobacteria and Actinobacteria. Proteobacteria, one of the most functionally diverse bacterial phyla, contribute to carbon fixation, organic matter decomposition, and plant–microbe symbioses, thereby enhancing nutrient acquisition (Peter et al., 2011; Wongkiew et al., 2022). Actinobacteria, in turn, degrade recalcitrant compounds, suppress soil pathogens, and facilitate nutrient turnover (Zhang et al., 2020; Rousk and Bååth, 2011). In parallel, fungi also play a crucial role in long-term ecosystem functioning by decomposing lignin and other recalcitrant organic compounds, stabilizing soil organic carbon through oregano–mineral associations, and enhancing nutrient cycling via mycorrhizal symbioses. Such contributions highlight their importance in sustaining nutrient turnover and carbon storage over extended timescales. In our study, their stable functional traits ultimately drove soil EMF under invasion conditions. These findings support our third hypothesis by demonstrating that community resistance mediated by core bacterial taxa plays a central role in sustaining soil EMF under invasion pressure. Taken together, they highlight the importance of core bacterial groups as ecological stabilizers that safeguard multifunctionality in invaded ecosystems.

The structural equation modeling further clarified the cascading pathway in which *A. philoxeroides* invasion and associated changes in soil properties significantly influenced soil EMF through community resistance mediated by core bacterial taxa (Figure 4). Under invasion pressure, core bacterial groups may strengthen resistance via two complementary mechanisms. First, their ability to utilize diverse carbon and nitrogen sources sustains metabolic activity under fluctuating resources, buffering community functioning against nutrient imbalances. Second, their high connectivity within microbial symbiotic networks facilitates resource redistribution, stabilizing both bacterial and fungal communities. This mechanism aligns with previous findings that cooperative interactions among dominant bacterial groups mitigate disturbance impacts on microbial structure and function (Wang et al., 2021). Moreover, core bacterial taxa were more responsive to environmental change than core fungal taxa, likely because fungi often exhibit narrower niches and slower turnover rates, limiting their capacity to rapidly compensate for functional losses (Jing et al., 2015; Xue et al., 2023). Ultimately, bacterial community resistance exerted a direct and significant positive effect on soil EMF, highlighting the pivotal role of core bacterial groups as ecological stabilizers in maintaining EMF under invasion. Our results suggest that core microbial taxa can serve as effective bio indicators of soil stability under invasion, offering a practical tool for monitoring belowground resilience. Their consistent contribution to soil EMF also highlights them as potential targets for microbial interventions, such as inoculation or soil amendments, to enhance resistance and accelerate ecosystem recovery. Integrating core microbial taxa into land management frameworks may therefore improve the prediction and restoration of soil functions in invaded ecosystems.

## 5 Conclusion

The invasion of *A. philoxeroides* significantly enhanced soil EMF at specific sites, such as Xiaoxian and Hefei, mainly driven by N_Mfa and P_Mfa, respectively. Integrating microbial resistance analysis, co-occurrence networks, and structural equation modeling, we found that bacterial communities exhibited stronger resistance than fungi, and this resistance was largely mediated by core bacterial taxa, including Proteobacteria and Actinobacteria. These taxa function as ecological stabilizers buffering invasion-induced disturbances and sustaining soil EMF. Notably, bacterial resistance exerted a direct and significant positive effect on EMF, highlighting its critical role in regulating EMF under invasion pressure. These findings, through cross-regional comparisons and comparative analyses of bacteria and fungi, have provided new mechanistic insights into the microbial basis of ecosystems’ responses to biological invasions, and it provides information for the control of invasive species, microbial inoculation or soil remediation measures, and at the same time offers a basis for predicting and managing the situation of soil electromagnetic fields in the context of future global changes. This study is based on a single sampling campaign, and future temporal monitoring would help capture seasonal dynamics and further refine predictions of soil EMF under invasion.

## Data Availability

The raw sequence data reported in this paper have been deposited in the Genome Sequence Archive (Genomics, Proteomics & Bioinformatics 2025) in National Genomics Data Center (Nucleic Acids Res 2025), China National Center for Bioinformation / Beijing Institute of Genomics, Chinese Academy of Sciences (GSA: CRA032627 and GSA: CRA031326) that are publicly accessible at https://ngdc.cncb.ac.cn/gsa.
