# Supplementary material for: Soil core microbial taxa maintain community resistance to drive soil ecosystem multifunctionality under Alternanthera philoxeroides invasion
Source: Front Microbiol. 2025 Nov 6;16:1707273. doi: 10.3389/fmicb.2025.1707273 (PMC12633765; doi:10.3389/fmicb.2025.1707273)
Supplement: Supplementary file 1 [file Table_1.DOC]

| Geographic information and site descriptions of sampling locations. | | | | | |
| --- | --- | --- | --- | --- | --- |
| Sample plot | latitude | Longitude | Annual mean precipitation(mm) | Annual mean temperature(℃) | Soil type |
| XiaoXian | 34°10′28″N | 116°56′51″E | 854 | 14.3 | sandy soil |
| Hefei | 31°45′31″N | 117°18′47″E | 1000 | 15.7 | yellow-brown soils |
| Anqing | 30°51′25″N | 116°21′11″E | 1427 | 14.5 | mountainous meadow soil |
| Tongling | 31°0′27″N | 117°58′30″E | 1412 | 15.9 | yellow-brown soil; yellowish-red soil |
| Huangshan | 29°40′18″N | 118°16′09″E | 1706 | 16.1 | red soil |
